# Computational fluid dynamics simulation experimental verification and analysis of droplets deposition behaviour on vibrating pear leaves

**DOI:** 10.1186/s13007-022-00914-x

**Published:** 2022-06-11

**Authors:** Yubin Cao, Te Xi, Lujiang Xu, Wei Qiu, Hongbin Guo, Xiaolan Lv, Chao Li

**Affiliations:** 1 College of Engineering/Key Laboratory of Intelligent Equipment for Agriculture of Jiangsu Province, Nanjing Agricultural University, Nanjing, 210031 China; 2 Jiangsu Academy of Agricultural Sciences, Nanjing, 210014 China

**Keywords:** Leaf vibration, Surface properties, Deposition behaviour, Simulation

## Abstract

**Background:**

The interaction between canopy and droplets is very important in the process of crop spraying. During the actual air-assisted application process, air-mist flow inevitably disturbs the leaves before droplets reaching them, which will also affect the final deposition state of the droplets on the leaf. Currently, researches on the interaction between droplets and the target leaf surface mainly focuses on the deposition behaviour on the surface of stationary target leaves rather than the dynamic leaves. Therefore, the deposition characteristics after the collision between the droplets and dynamic leaves are important for practical application and worth further study.

**Results:**

Computational fluid dynamics simulations were performed to characterise the surface roughness, contact angle, and mechanical vibration. The interaction platform between the droplet and the vibrating pear leaf was built for experimental verification under laboratory conditions. The simulation results are in good agreement with the experimental results, which revealed the main reason for the droplet spreading and sliding was the inertial force generated by the relative velocity. It also indicated that the pear leaf vibration can improve the deposition of low-velocity and small droplets, which is different from that of static pear leaves.

**Conclusion:**

The deposition effect of droplets in vibrating pear leaves was investigated. This study also provides a simulation method for the collision between a vibrating leaf and moving droplets, and provides reference for the study of droplet deposition characteristics under the vibration of fruit trees.

## Background

To prevent yield loss caused by diseases and pests, plant protection operations such as spraying pesticides are necessary preventive measures in the planting process for fruit trees. Fruit trees are sprayed with pesticides 8–15 times in a year, accounting for 30% of the total workload of orchard management. Frequent pesticide applications also cause environmental pollution, pesticide residues, and increase in labour cost [1]. Air-assisted spraying technology has been widely recognised for its precision, portability, and efficiency. From the development of the first orchard air spray machine using axial flow fan in 1949 to now, various orchard air-assisted sprayers were developed to improve the spray deposition [2–6]. As is known, air-assisted spraying is a complex process. Droplets are transported by assisted airflow after atomisation, and a series of deposition behaviours occur following collision with plant leaves disturbed by airflow. In the entire process, the interaction between droplets and fruit tree leaves is a crucial link that determines whether droplets can be deposited and spread effectively. Studies have shown that although droplets can reach a target, a series of phenomena such as rebound, splashing, and drift will occur after the droplets collide with a leaf. More than 50% of droplets are difficult to deposit on leaves [7].

In order to improve the efficiency of the spraying process, the interaction between droplets and leaves has been extensively studied. Because the diameter of droplets is small, high-speed camera is often used to describe the collision process of droplets impacting a target surface [8, 9]. In the process of droplets impacting plant leaves, fragmentation, rebound, splashing and spreading will occur, affecting the final deposition amount of droplets on a leaf surface [10–13]. The impact factors between droplets and plant leaves mainly include droplet type, droplet viscosity, droplet size, impact velocity, impact angle, contact angle, surface type, surface geometry, and surface roughness [14–16]. All these factors affect the dynamic characteristics of droplets after collision with plant leaves. For example, Dorr et al. developed a turbulence probability model for the interaction between droplets and plant surfaces to improve the deposition of droplets on leaves [17]. Nairn et al. found that the hairy structure on a leaf surface was conducive to the splitting and spreading of droplets, which could improve the deposition effect of droplets [18]. Puente et al. found that the difficult degree of wetting of droplets on the leaf surface, that is, the contact angle of droplets on the leaf surface affected the deposition effect of droplets on the leaf surface [19]. Massinon et al. studied a logistic regression model to predict the influence of droplet surface characteristics on deposition behaviour [20]. These experiments and findings provided good ideas and methods for future research on deposition. However, most researchers focused on the deposition behaviour on the surface of stationary target leaves. Air-mist flow inevitably disturbs the leaves before reaching them in the actual air-assisted application process. This disturbance will cause these parameters, such as impact velocity, impact angle, contact angle, to change, affecting the deposition state of droplets on leaves. Therefore, the deposition characteristics after the collision between the droplets and dynamic leaves need to be further studied.

In recent years, numerical simulations using computational fluid dynamics (CFD) have been widely used due to the unique advantages of low cost, fast velocity, complete data, and can complement the limitations of the field experiment [21, 22]. Various CFD models, such as the Lagrange model for studying droplet trajectory, have been widely applied to simulate the factors that are difficult to control in field experiments, particularly in the optimisation of fan internal structure, airflow field distribution, droplet deposition, and drift [23–26]. However, considering the complexity of CFD model, there have been only a few studies on the interactions between pesticide flows and leaves [10, 27]. In addition, in the limited research, only immobile leaves have been considered. As the droplets reach the canopy, the airflow disturbs the leaves and has a fundamental impact on the droplet deposition behaviour. Comprehensive CFD simulations are necessary for understanding the complex processes of droplet-leaf deposition behaviour by establishing the simulation conditions of dynamic leaves and then simulating the scene closest to the actual situation.

In the many past studies, only the droplet deposition characteristics of the static leaves are considered, while the deposition characteristics of the dynamic leaves disturbed by the airflow in the actual spraying process are ignored. There is a lack of research on the dynamic characteristics of the disturbed leaves and the deposition behaviour of the droplets on the dynamic leaves. In this study, to explore the influence of pear leaf vibration on droplet collision and deposition, a collision mechanics model and sliding mechanics model were established. Accordingly, this study adopted the method of combining CFD and practical experiments to simulate the deposition behaviour after the collision between droplets and leaves. A theoretical model of the droplet deposition behaviour in the leaf vibration state was established. The influence of leaf vibration on the droplet deposition behaviour and its internal mechanism was investigated, providing a reference for the study of droplet deposition characteristics under the vibration of fruit trees. This study enriches the basic theory of the atomisation mechanism and application of orchard spraying technology, providing a theoretical basis for spraying parameter settings and sprayer design.

### Materials and methods

#### Experimental platform

**Fig. 1:**
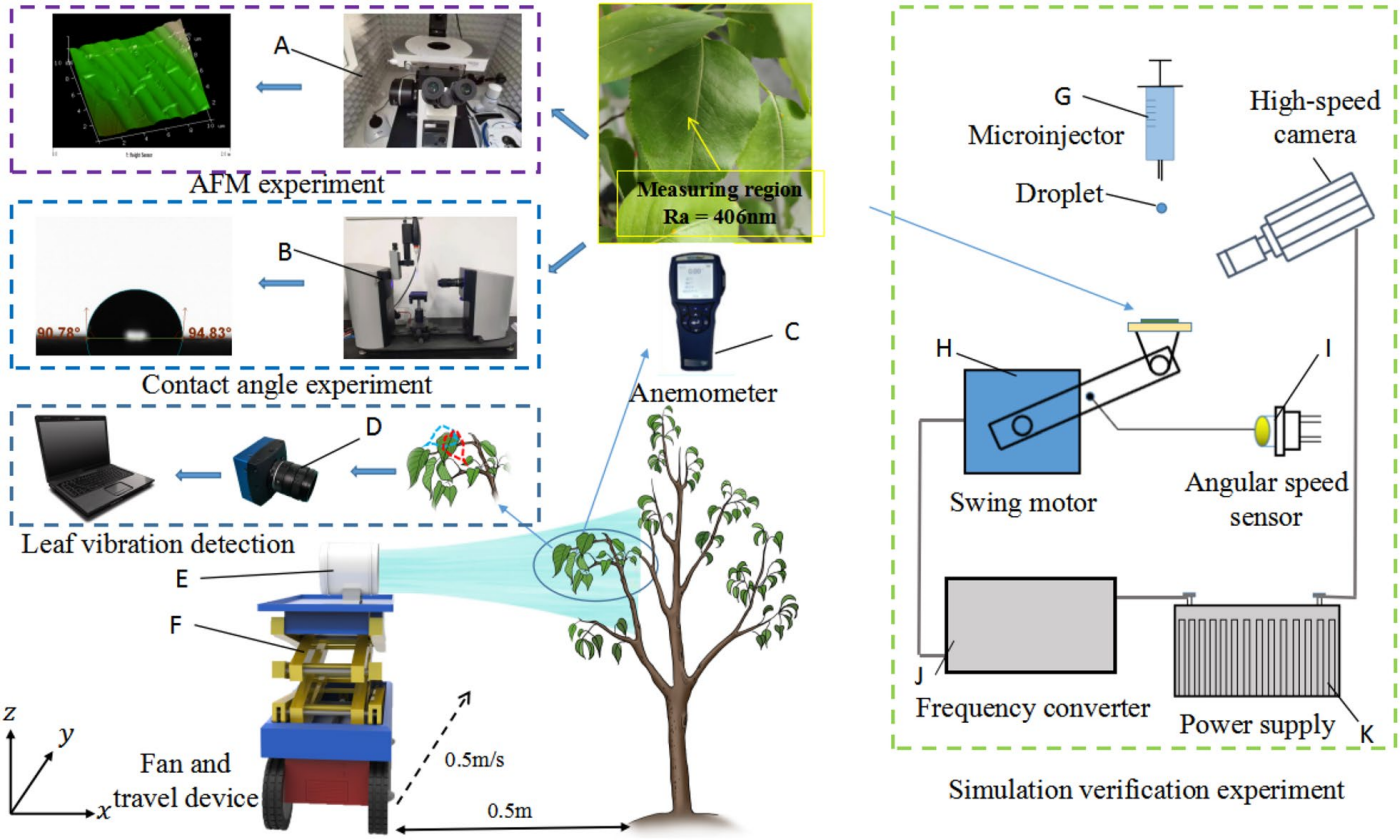
Experimental flowchart. **A** atomic force microscopy; **B** contact angle measuring instrument; **C** anemometer; **D** high-speed camera; **E** fan; **F** lifting gear; **G** microinjector; **H** swing motor; **I** angular speed sensor; **J** frequency converter; **K** power supply

As shown in Fig. 1, to accurately conduct CFD simulation and verify the CFD simulation process, the experiment included the atomic force microscopy (AFM) apparatus, contact angle measurement platform, pear leaf vibration detection platform, and interaction experimental platform between droplets and pear leaves. AFM (BRUKER; Brock Technology Co., LTD; scanning surface roughness of pear leaves) was used as the main experimental instrument for the AFM platform. The main experimental instrument for the contact angle measuring platform was the contact angle measuring. The pear leaf vibration detection platform includes a fan controlled by a frequency converter (diameter of 150 mm; to control incoming flow velocity, the wind velocity at the outlet is 0–15 m/s), a TSI9545 hot wire anemometer (measurement error of incoming flow velocity of ± 0.025 m/s), a walking system (fixed walking velocity of 0.5 m/s), lifting device (lifting distance of 0.2–2.1 m), and a high-speed camera (ACS-1 M60; Nac Image Technology, Japan; shooting frequency of 110 fps). The interaction experimental platform between droplets and pear leaves included a microinjector (0.1–2 μL), high-speed camera, swing motor (motor velocity of 0–6400 RPM), angular velocity sensor, frequency converter, and power supply.

### Determining the pear leaf parameters

#### Experimental materials

In April 2021, the temperature was approximately 20 °C and the humidity was 35–50%. Fresh pear tree branches and leaves were collected at the horticultural plantation base of Nanjing Agricultural University, Nanjing, Jiangsu Province. The experimental tree was a seven-year-old crown pear, and the main branch leaf was natural. Sufficient fresh leaf samples were taken from the top, bottom, and side of the crown, and the time from sampling to experiment was controlled within 12 h.

#### Surface parameters of pear leaves

The surface morphology of pear leaves is complex and has a significant influence on droplet impact dynamics [28]. As shown in Fig. 1, to better characterise the surface morphology of pear leaves in the CFD simulation process, AFM was used to scan the leaves to obtain their surface roughness. AFM is the contact mode. The measurement error is less than 0.1 nm. Ten leaves were randomly selected from the sample and scans were repeated three times for each leaf to obtain good accuracy. The results showed that the average surface roughness of the pear leaves was 406 nm. To set the interaction effect between the droplet and leaf surface in the CFD simulation process, as shown in Fig. 1, the contact angle of the droplet on the pear leaf surface in the static state was measured using a contact angle measuring instrument. The measurement was repeated three times for each of the above ten leaves. The contact angle of the pear leaf surface was found to be $92.58^\circ \pm 1.80^\circ$. The criteria for judging surface hydrophilic is as follows: The surface with a contact angle less than 90° is hydrophilic, and that with a contact angle greater than 90° is hydrophobic [29]. Therefore, the pear leaves are hydrophobic.

#### Vibration parameters of pear leaves

Ten intact pear leaves were selected for each sample. The average petiole characteristic length ${L}_{\mathrm{m}}/{S}^{0.5}$ of pear leaves was 20 [30]. Here, ${L}_{\mathrm{m}}$ is the maximum petiole length, $S$ is the average sectional area of the petiole, the average leaf length is 80 mm, and the average leaf thickness is 1 mm. The centre of the leaf was considered as the mark point, and the rotation radius was 10 cm. The pear leaves drooped naturally and faced the fan outlet. The running velocity of the fan was 0.5 m/s. The outlet wind velocity was adjusted by the frequency converter, and the inflow velocity was controlled using an anemometer at 5, 6, 7, 8, 9, and 10 m/s. The angle between the shooting direction of the high-speed camera and the direction of the inflow velocity was set to 90°. The movement of pear leaves in the elastic limit under the action of the wind is divided into three stages: compression, high-frequency small-amplitude vibration, and rebound. The displacement and angle changes in each stage of the pear leaf vibration video were captured to realize the amplitude, swing angle, and average angular velocity range of pear leaf vibration. The experiment was repeated three times at each inflow velocity for a total of 180 iterations. The vibration parameters of the pear leaves at different stages were obtained and are listed in Table 1.

**Table 1:** Vibration parameters of pear leaves

Inflow velocity(m/s)	5	6	7	8	9	10
Compression stage						
Amplitude (cm)	3.1 ± 0.7	5.0 ± 0.4	6.0 ± 0.6	7.1 ± 0.8	8.1 ± 0.8	10.1 ± 1.3
Swing angle (°)	39.5 ± 4.5	59.5 ± 2.9	77.5 ± 3.7	103.0 ± 5.7	130.5 ± 6.1	157.0 ± 9.8
Average angular velocity (rad/s)	4.9 ± 0.6	5.2 ± 0.3	6.8 ± 0.3	9.0 ± 0.5	11.4 ± 0.5	13.7 ± 0.9
High-frequency small-amplitude vibration stage						
Amplitude (cm)	2.2 ± 0.4	2.6 ± 0.4	1.9 ± 0.2	1.8 ± 0.2	1.8 ± 0.3	1.6 ± 0.2
Swing angle (°)	32.5 ± 6.2	37.5 ± 6.0	27.8 ± 2.7	26.5 ± 2.9	22.5 ± 2.6	22.0 ± 2.0
Average angular velocity (rad/s)	2.9 ± 0.6	3.9 ± 0.7	3.8 ± 0.4	4.5 ± 0.7	4.9 ± 0.7	5.7 ± 0.9
Rebound stage						
Amplitude (cm)	3.2 ± 0.7	4.8 ± 0.6	5.9 ± 0.5	7.0 ± 0.8	7.8 ± 0.8	10.0 ± 1.1
Swing angle (°)	39.5 ± 3.7	60.5 ± 2.9	76.5 ± 3.4	100.5 ± 5.3	129.5 ± 6.1	157.5 ± 11.8
Average angular velocity (rad/s)	3.4 ± 0.3	5.3 ± 0.3	6.6 ± 0.3	8.8 ± 0.5	11.3 ± 0.5	13.7 ± 1.0

The data in the table are average values ± standard deviation

### CFD simulation

#### CFD model

Turbulence is an irregular, multiscale, and structured physical phenomenon. It is a state of flow, not a fluid property. Laminar flow is symmetric, whereas turbulent flow is asymmetric. It is usually a three-dimensional unsteady flow and has a strong diffusion and dissipation ability. In terms of the physical structure, turbulence is a flow formed by the superposition of vortices and rotating structures at different scales. The size and directional distribution of these vortices were random. Regarding turbulence model theory, or turbulence model for short, turbulence motion is almost infinite in physics and nonlinear in mathematics, which makes it difficult to solve the turbulence problem.

The RNG *k*-*ε* model is derived from statistical methods and is similar to the standard *k*-*ε* model in form, but with the following improvements: a condition is added to the equation, which effectively improves the accuracy of the flow; The standard *k*-*ε* model is a high-Reynolds-number model, while the RNG theory considers a low Reynolds number. These characteristics make the RNG *k*-*ε* model more reliable and accurate in fluid flow than the standard *k*-*ε* model. The *RNG k*-*ε* turbulence model is given by [31]:

1 $$\frac{\partial (\rho k)}{\partial t}+\frac{\partial (\rho k{u}_{i})}{\partial {x}_{i}}=\frac{\partial }{\partial {x}_{i}}\left({\alpha }_{k}{\mu }_{eff}\frac{\partial k}{\partial {x}_{j}}\right)+{G}_{k}+{G}_{b}-\rho \varepsilon -{Y}_{m}+{S}_{k},$$

2 $$\frac{\partial \left(\rho \varepsilon \right)}{\partial t}+\frac{\partial \left(\rho \varepsilon {u}_{i}\right)}{\partial {x}_{i}}=\frac{\partial }{\partial {x}_{i}}\left({\alpha }_{\varepsilon }{\mu }_{eff}\frac{\partial \varepsilon }{\partial {x}_{j}}\right)+{C}_{1\varepsilon }^{*}\frac{\varepsilon }{k}\left({G}_{k}+{C}_{3\varepsilon }{G}_{b}\right)-{C}_{2\varepsilon }\rho \frac{{\varepsilon }^{2}}{k}-{R}_{\varepsilon }+{S}_{\varepsilon }.$$

Multiphase flow is a fluid movement in which two or more substances of different phases coexist. When calculating the gas–liquid–solid three-phase interaction, the volume of fluid (VOF) is a classic method for dealing with interface problems. Based on the volume fraction of the gas–liquid phase in the flow field at different times, the gas–liquid distribution was constructed to form the gas–liquid interface. However, when selecting the VOF model, laminar flow and large eddy simulations cannot be used in the turbulence model. The volume fraction continuity equation is [32]:

3 $$\frac{\partial {\alpha }_{1}}{\partial t}+u\cdot \nabla {\alpha }_{1}=0,$$

4 $$\frac{\partial {\alpha }_{2}}{\partial t}+u\cdot \nabla {\alpha }_{2}=0.$$

The continuity equation is as follows:

5 $$\frac{\partial \rho }{\partial t}+\nabla \cdot \left(\rho u\right)=0.$$

The momentum equation is given as:

6 $$\frac{\partial \rho u}{\partial t}+\left(\rho u\cdot \nabla \right)u=-\nabla \rho +\nabla \cdot \left[\mu \left(\nabla u+\nabla {u}^{T}\right)\right]+\rho g+F,$$

where ${\alpha }_{1}$ and ${\alpha }_{2}$ are the volume fractions of different components (${\alpha }_{1}+{\alpha }_{2}=1$), $\rho$ is density, $\mu$ is the dynamic viscosity coefficient, $t$ is time, $u$ is velocity, $p$ is static pressure, $g$ is acceleration due to gravity, and $F$ is the equivalent volume force of surface tension. The overall property of a substance is determined by the percentage of components of each phase in the controlling body of each part, and the relevant calculation expression is given by:

7 $$\rho ={\alpha }_{1}{\rho }_{1}+{\alpha }_{2}{\rho }_{2},$$

8 $$\mu ={\alpha }_{1}{\mu }_{1}+{\alpha }_{2}{\mu }_{2},$$

where ${\rho }_{1}$ and ${\rho }_{2}$ are the densities of the liquid and gas, respectively, and ${\mu }_{1}$ and ${\mu }_{2}$ are the dynamic viscosity coefficients of the liquid and gas, respectively. In addition, when the dynamic interface interaction between the leaf and the pesticide droplet is considered, the state equation of the liquid–gas mixture and turbulence equation is adopted. The specific formula for the state equation is [33]:

9 $$P=\frac{{R}_{g}\rho T{\sum }_{k}{Y}_{K}}{{W}_{k}},$$

10 $$E={\sum }_{k}{Y}_{k}\left({c}_{vk}T+{h}_{ok}\right)+\frac{{\underset{u}{\to }}^{2}}{2}+k,$$

where $E$ is the gas energy, $k$ is the turbulent kinetic energy, ${W}_{k}$ is the molar mass of the *k*th gas component, ${h}_{ok}$ is the specific chemical energy, ${c}_{vk}$ is the specific heat capacity, ${Y}_{k}$ is the mass concentration of the *k*th gas component, $T$ is the gas temperature, and ${R}_{g}$ is the universal gas constant. For details of this equation, please refer to the work of Menter [31].

#### Simulation conditions

**Fig. 2:**
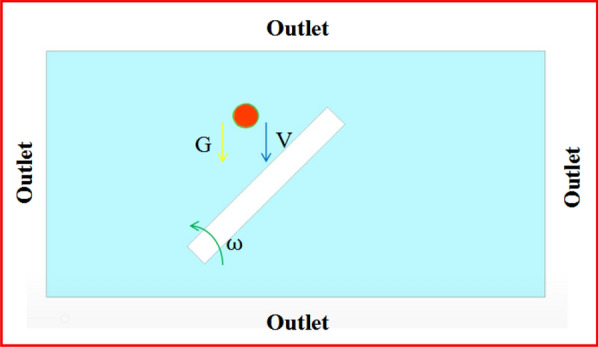
Region of dynamic droplet impact

ANSYS Fluent software was used for the simulations. Figure 2 shows the simulation geometry domain. For the boundary condition, pressure outlets were around. The leaf was simplified as a long rectangle, and the leaves were surrounded by air. The mesh size was 0.05 mm, the number of nodes was 77,387, and the mesh number of droplets after local initialisation was 643. To simplify the calculation of multiphase flow, structured grids were selected. To improve the calculation accuracy, the grid division method was unified in the fluid domain.

In the solver, the droplet density was set to ${10}^{3}\mathrm{ kg}/{\mathrm{m}}^{3}$, and the viscosity was $2.98\times {10}^{-3}\mathrm{ pa}\cdot \mathrm{s}$. The acceleration due to gravity is $9.81\mathrm{ m}/{\mathrm{s}}^{2}$, the surface roughness is 406 nm, and the contact angle is $92.58^\circ \pm 1.80^\circ$. The $VOF$ and $RNG$ *k*-ε turbulence models were used. Two phases of air and water were used in the VOF model. The volume fraction parameter is explicit, the Courant Number is 0.25, the volume force formula for implicit body force is enabled, the global option enables surface tension modelling, and the continuum surface force and adhesion model. Air was used as the primary phase. The surface tension coefficient of the interaction between water and leaf surface was set to $0.072\mathrm{ N}/\mathrm{m}$. It can be seen from the data in Table 2. The $RNG$ *k*-ε turbulence model was applied to the entire fluid domain to obtain accurate simulation results.

**Table 2:** Method and value of some parameters in the simulations

Method/parameter	Settings/value
Calculation type	Transient model
Calculation model	Volume of fluid
Grid size	0.05 mm
Gravity	9.81 m/s^2^
Operating temperature	293 K
Surface roughness	406 nm
CA	92.58°
Surface tension	0.072 N/m
Water density	10^3^ kg/m^3^
Water viscosity	2.98 × 10^–3^ pa·s
Time step	0.00001 s
Step number	1000

### CFD simulation verification

As shown in Fig. 1, in the interactive experimental platform of droplets and pear leaves, a microinjector was used to set 1 μL. The droplet size was 1,242 μm. Droplets collided with static leaf centres at 0°, 30°, 45°, and 60° under the action of gravity. The velocity of droplets reaching the leaf surface was set at 2 m/s by changing the droplet height to the leaf surface. A high-velocity camera was used to capture the impact behaviour of the droplets. The vibration platform was used to set the distance between the rotating centre and the leaf centre to 10 cm in the dynamic experiment. Pear leaves were rotated with angular velocities of 3, 6 and 12 rad/s, and the leaf angles were 0, 30°, 45°, and 60°, respectively, at the moment of impact. The angular velocity sensor was set to control the angular velocity of the rotational motion, and the collision position was always at the centre of the leaf by controlling the collision time difference. A high-velocity camera was used to record the entire process of droplet collision and deposition in 120 experiments. The longitudinal spreading factor $\beta ={D}_{s}/{D}_{0}$ was used as the index of the droplet deposition range [34]. Here, ${D}_{0}$ is the initial droplet diameter and ${D}_{s}$ is the spreading diameter along the horizontal direction of the leaf surface. When the final spreading factor was 1, the droplet did not spread. When the spreading factor was greater than 1 for the first time, the critical velocities in the horizontal and vertical directions were recorded. In this study, the accuracy of the model was evaluated using the root mean squared error (RMSE), as shown in Table 3. The same test parameters were selected and executed in the simulation, and the data from both (actual test and simulation) were compared and analysed to verify the feasibility and accuracy of CFD simulation.

**Table 3:** The deviation between simulation value and true value of $\beta$ based on the velocity of 1-μL droplet

Leaf angles	Angular velocity	RMSE
0°	3	0.043
	6	0.033
	12	0.032
30°	3	0.029
	6	0.043
	12	0.033
45°	3	0.040
	6	0.036
	12	0.048
60°	3	0.037
	6	0.041
	12	0.035

### CFD analysis of droplet deposition behaviour of three sizes under vibration condition

The validated CFD model was used to analyse droplet deposition behaviour of the three size droplets. Dynamic grid technology was used in the leaf motion simulation process, turning on spring smoothing and mesh reconstruction. The spring fairing method was the diffusion method, and the mesh was redevised into open local units. The dimension adjustment function was applied to set the minimum and maximum length scales to 0.015 and 0.05 mm, respectively, and the maximum element skewness was 0.8. The grid repartitioning interval was one time step to ensure the quality of the grid repartitioning interval of 1 time step. Combined with the vibration parameters obtained from the vibration test of pear leaves, the vibration angular velocity of pear leaves at a rated wind velocity is within 15 rad/s. Therefore, the rotation angular velocity was set to 3, 6, and 12 rad/s by importing the profile. The corresponding inflow velocities were 5, 6–7, and 9–10 m/s.

According to the actual situation, the fluid outside should have the same conditions as the fluid domain, and the gauge pressure at the pressure outlet should be set to 1 atm (101.325 kPa). The numerical simulation method is the first-order model of the finite difference method, and a simple algorithm was used for the simulation. After initialising the computational domain, the droplet was initialised locally. The initial velocity and impact angle of the droplet were set, and the volume fraction of water was set to 1. After setting the time step to ${e}^{-5}$ and the number of time steps to 1,000, the calculation started. When the single simulation calculation time was less than 8 h, the minimum residual differences of all items except energy were less than 10^–4^, and the minimum residual values of energy were less than 10^–6^. The spreading process and final longitudinal spreading range of the droplets were recorded.

Three different sizes (576, 984, and 1242 μm) of droplets were set. The velocity range of droplets reaching the leaf surface was from 0 to 5 m/s, with the gradient of 0.5 m/s. Initial angles of pear leaves were selected as 0°, 30°, 45°, and 60°. The angular velocities of leaves were set at 3, 6, and 12 rad/s, respectively. A total of 360 experiments were conducted in this study.

## Results and discussion

### Theoretical analysis

#### Mechanical model of droplet impact on vibrating horizontal leaf

**Fig. 3:**
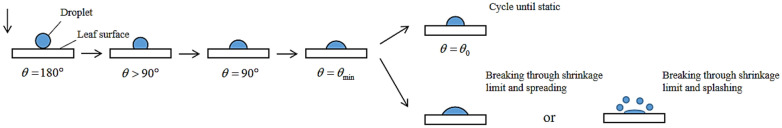
Collision process between a droplet and a leaf

The effect of vibration on the dynamics of droplets on the leaf was investigated via establishing the mechanical model, which lays a theoretical foundation for analysis of droplet deposition behaviour in the subsequent experiment. Because the quality of droplets is comparatively small, the deformation of pear leaves in the process of droplet impact was not considered. The inertial force and surface tension are considered the main factors in the process of droplet collision, and a dynamic equilibrium relationship exists between them in the case of static deposition. In the droplet collision process, the volume of the droplet was constant (the volume of the droplet used in the calculation was 1 μL). In vertical collision with the leaf surface, the calculation of the droplet volume was based on the volume formula for the segment. Different surface tensions and inertial forces depend on the leaf surface roughness, evaporation, and liquid viscosity. Surface tension is the force exerted by a liquid, such as water, to shrink the surface as much as possible. Surface tension causes droplets to shrink inward, making it difficult for droplets to spread out. As shown in Fig. 3, the contact angle change in droplets that can be eventually deposited in the collision process varies from 180° to 90°, then to ${\theta }_{\mathrm{min}}$, and finally to ${\theta }_{0}$. The results of the collision between the droplets and pear leaves can be deposition, spreading, and splashing.

**Fig. 4:**
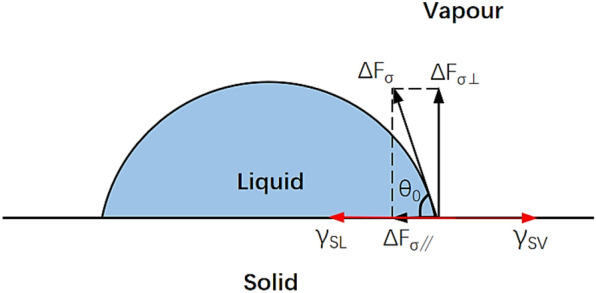
Surface tension and interface tension between solid and liquid

As shown in Fig. 4, it can be observed from Young’s equation that:

11 $${\gamma }_{sv}={\gamma }_{sl}+{\gamma }_{lv}cos\theta ,$$

where ${\gamma }_{sv}$ is the interfacial tension between the solid and gas, ${\gamma }_{sl}$ is the surface tension between the solid and liquid, and ${\gamma }_{lv}$ is the interfacial tension between the gas and liquid. Furthermore, ${\gamma }_{lv}$ is equal to $\Delta {F}_{\sigma }$, and ${\theta }_{0}$ is the static contact angle. The surface tension ${F}_{\sigma }$ is expressed as follows:

12 $${F}_{\sigma }=\sum \Delta {F}_{\sigma }=\sigma l=2\pi r\sigma ,$$

where $l$ is the length of the liquid phase line and $r$ is the spreading radius of the droplet. The surface tension was decomposed in the vertical and horizontal directions.

13 $$\mathrm{F}\sigma \perp =2\pi r\sigma \cdot sin\theta$$

14 $$F\sigma \text{//}=2\pi r\sigma \cdot cos\theta$$

**Fig. 5:**
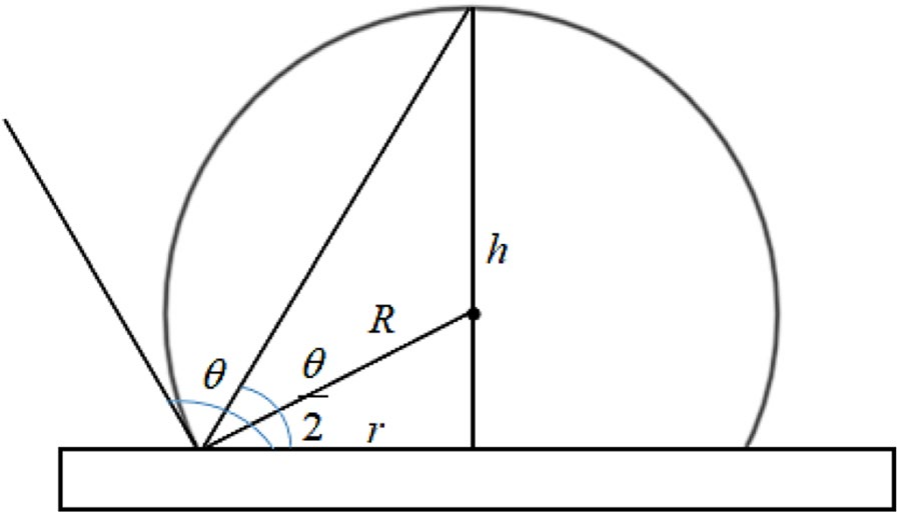
Mathematical model of collision process

For the droplet collision process, the following relationship exists in the mathematical model shown in Fig. 5:

15 $$\mathrm{tan}\frac{\theta }{2}=\frac{h}{r},$$

16 $$sin\theta =\frac{r}{R},$$

where $h$ is the height of the droplet and $R$ is the radius of the droplet. Based on the volume formula of the segment,

17 $$V=\frac{1}{3}\pi {h}^{2}\left(3R-h\right).$$

The relationship between the spreading radius and contact angle was obtained with the volume unchanged.

18 $$r=\sqrt[3]{\frac{3Vsin\theta (1+cos\theta )}{\pi (1-cos\theta )(2+cos\theta )}}.$$

Substituting (18) into (13) and (14) obtains:

19 $${F}_{\sigma \perp }=2\pi \sigma \cdot sin\theta \sqrt[3]{\frac{3Vsin\theta (1+cos\theta )}{\pi (1-cos\theta )(2+cos\theta )},}$$

20 $${F}_{\sigma //}=2\pi \sigma \cdot cos\theta \sqrt[3]{\frac{3Vsin\theta (1+cos\theta )}{\pi (1-cos\theta )(2+cos\theta )}.}$$

**Fig. 6:**
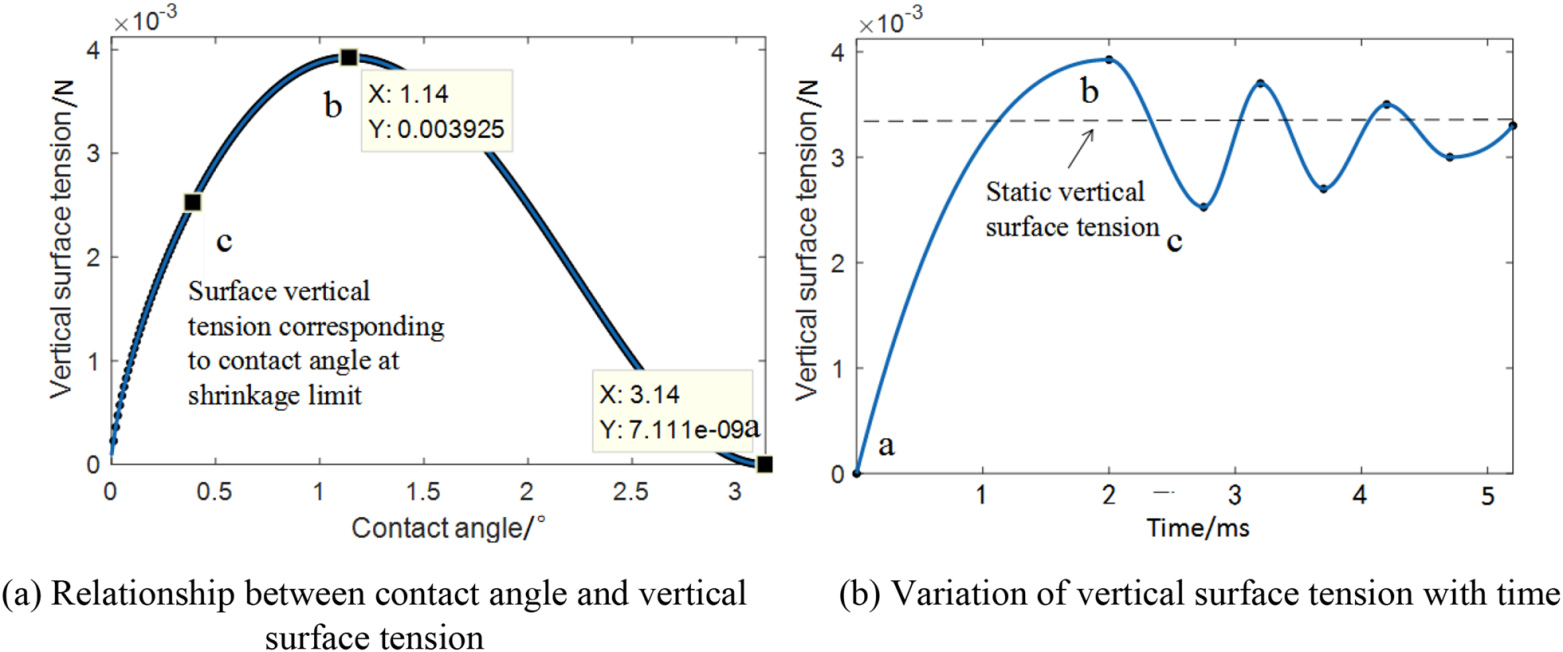
MATLAB fitting curve of vertical surface tension

According to the above surface tension formulas ((19) and (20)), MATLAB software was used to solve the equations and perform image processing, and the MATLAB fitting curve of vertical surface tension was obtained, as shown in Fig. 6.

**Fig. 7:**
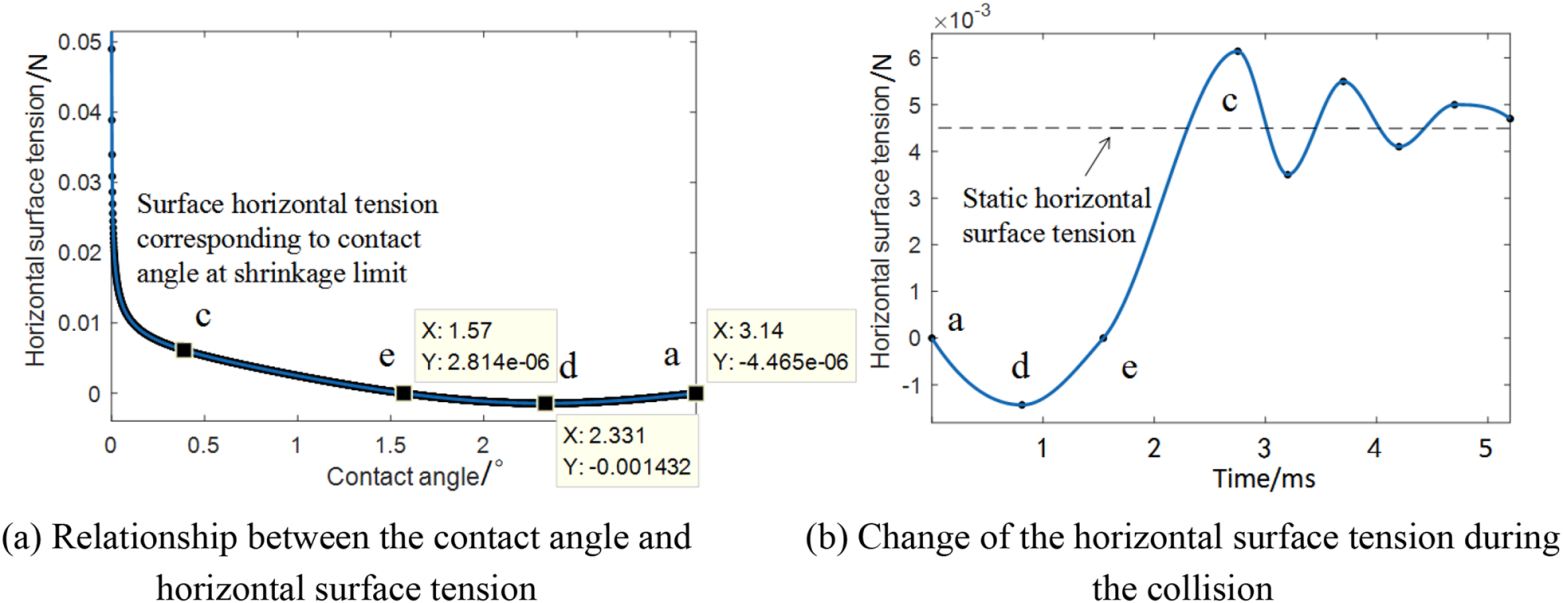
MATLAB fitting curves of the surface tension in the horizontal direction

Figure 6a shows the relationship between the contact angle and the vertical surface tension. Point a indicates the condition when the droplet first contacts the leaf. Point b represents the maximum vertical surface tension of the droplet during collision, and point c represents the vertical surface tension at the shrinkage limit. Figure 6b shows the variation in the vertical surface tension with time during droplet collision. As shown at the back end of point c, the vertical surface tension of the droplets tends to be static. In the droplet collision process, if the droplet breaks through point c while transitioning from b to c owing to the influence of force or motion, spreading of the droplet will increase by a certain amount in the original deposition range. The MATLAB fitting curve for horizontal surface tension is presented in Fig. 7.

Figure 7a displays the relationship between the contact angle and the horizontal surface tension. In Fig. 7b, unlike the vertical surface tension, the horizontal surface tension is outward in the direction of the a–e segment, and the droplets tend to spread outward at the initial collision time. In the e–c segment, the direction of the horizontal surface tension is inward, and the droplets tend to contract inward. Similarly, if the droplet breaks through the contraction limit of point c owing to the force or motion of the droplet, the droplet spreads. Simultaneously, the inertial force is introduced, given by:

21 $${F}_{I}=-ma=-m\frac{\Delta v}{\Delta t}.$$

The direction of the inertial force is opposite to that of the acceleration. In the droplet collision process, an obtuse angle exists between the direction of the inertial force and the surface tension. The inertial force causes the droplet to deform, whereas the surface tension hinders deformation. The relative velocity increased when the droplet collided with the dynamic horizontal leaf. However, in the case of a constant mass, the velocity change rate and impact time directly determine the magnitude of the inertial force. The impact time of droplets is quite short; thus, the vibration velocity of pear leaves can change the inertial force to a certain extent, leading to a difference in the droplet deposition behaviour.

In conclusion, when a droplet collides with a static leaf, the contact angle is directly related to the surface tension, and there are limits to the surface tension at the time of the collision. The vertical surface tension first increases to the maximum value, then decreases to the minimum value, and then fluctuates back and forth as the amplitude decreases. The horizontal surface tension first decreases to a minimum, then increases to a maximum, and then fluctuates back and forth with decreasing amplitude. Meanwhile, the vibration velocity of the pear leaves can change the magnitude of the inertial force. The change in inertial forces prevents the droplets from staying in place, destroying the droplet shrinkage limit and spreading. Therefore, the model explained below was designed.

#### Slip model of droplet impact on vibrating inclined leaf

**Fig. 8:**
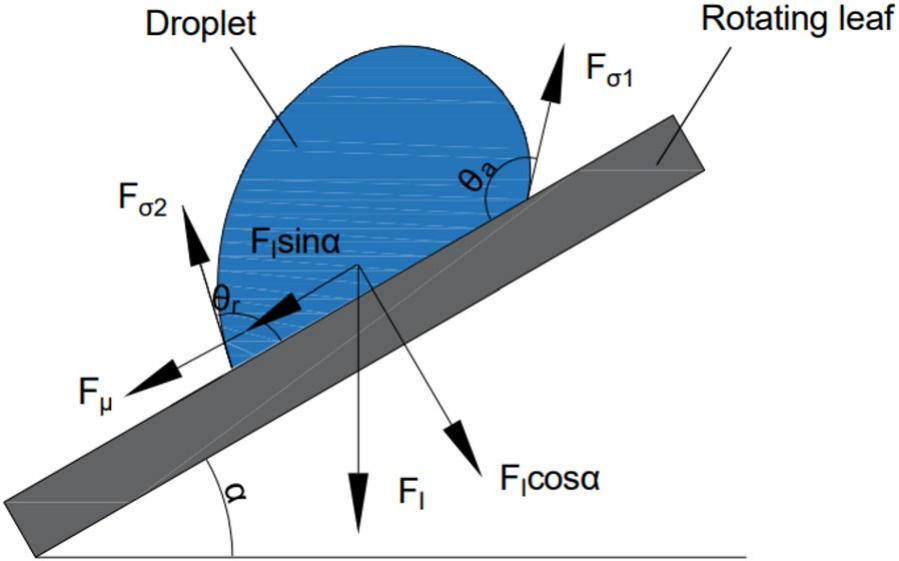
Force diagram of a droplet impacting a rotating leaf

When a droplet collides with an inclined wall, sliding in the horizontal direction becomes easy owing to the horizontal force. Therefore, the slip phenomenon at the time of the droplet collision was analysed. Figure 8 shows a force diagram of the droplet impacting the rotating leaf.

In Fig. 8, ${\theta }_{\mathrm{a}}$ is the advancing angle, $\Delta {F}_{\sigma 1}$ is the surface tension corresponding to the advancing angle, ${\theta }_{\mathrm{r}}$ is the retreating angle, $\Delta {F}_{\sigma 2}$ is the surface tension corresponding to the retreating angle, $\omega$ is the angular velocity of leaf rotation, and $\alpha$ is the leaf inclination angle at the moment of collision of the rotating leaf with the droplet. At the moment of droplet sliding, relative motion between the droplet and the leaf occurs; thus, a viscous force ${F}_{\mu }$ exists, which is calculated as:

22 $${F}_{\mu }=\mu \cdot \frac{dv}{dz}\cdot A,$$

where $\mu$ is the dynamic viscosity, $dv/dz$ is the fluid velocity gradient, and $A$ is the solid–liquid contact area. Droplet sliding is hindered by the viscous force. Meanwhile, because the contact angles of the two sides of the droplet are inconsistent, a difference in the transverse surface tension exists. Because the deformed droplet is a three-dimensional surface, the resultant force of the maximum transverse surface tension on the solid–liquid contact line generated by the difference in contact angle is ${F}_{t}$. Therefore, the conditions for the slip are as follows:

23 $${F}_{I}sin\alpha \ge {F}_{\mu }+{F}_{t},$$

where ${F}_{I}$ is mainly determined by $\Delta v/\Delta t$; the relative velocity difference $\Delta v$ is determined by the rotation angular velocity, collision position, and velocity of the droplet reaching the leaf surface; and the leaf inclination angle $\alpha$ determines the horizontal component of the inertial force. The value of ${F}_{\mu }$ is smaller in the process of small droplet slip; hence, the main factor that restrains the slip is ${F}_{t}$.

In summary, the inclination angle during collision divides the inertial force into vertical and horizontal directions. In the direction perpendicular to the inclined plane, it is similar to the collision between the droplet and the vibrating horizontal leaf. The inertial force increased with the angular velocity of the vibration, making it possible for the droplet to spread after breaking the shrinkage limit. The inertial force in the horizontal direction may also cause a slip. Therefore, two types of spreading caused by the collision between the droplet and the rotating leaf exist: sliding caused by the inertial force in the horizontal direction and spreading caused by the inertial force in the vertical direction.

### CFD simulation verification

**Fig. 9:**
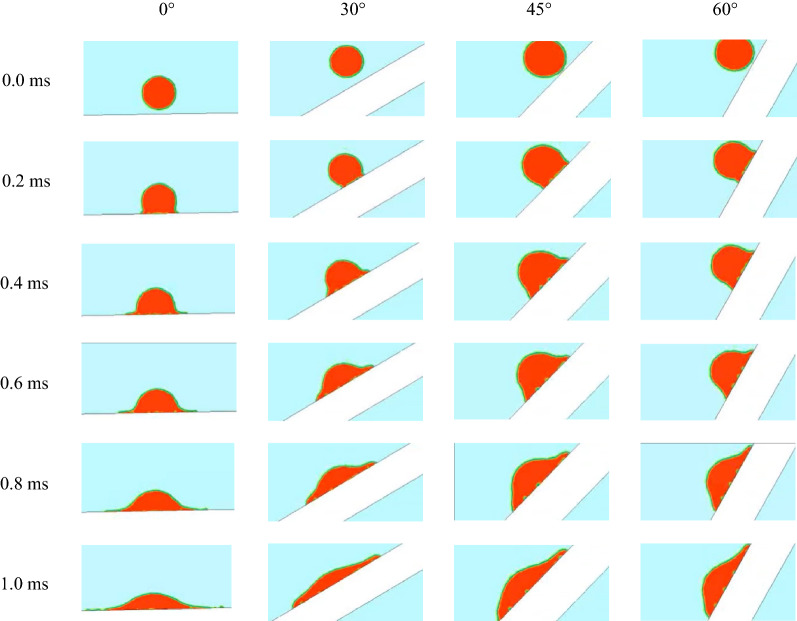
Simulated collision process of 1-μL droplet with initial velocity of 2 m/s and a pear leaf with angular velocity of 12 rad/s

In this study, a simulated collision between a 1-μL droplet with the velocity of 2 m/s and a pear leaf with the angular velocity of 12 rad/s was used as an example to illustrate the liquid phase change during the collision process. As shown in Fig. 9, the liquid distribution of the droplets on the $0^\circ$ inclined pear leaves extended from the centre to both sides. At 0.4 ms, owing to the high relative velocity of the droplet and the rotating pear leaf, the inertial force caused the droplet to break through the vertical shrinkage limit. The liquid film at the edge of the droplet deformed, and the droplet began to spread out. When the collision energy was lost at 1 ms, the relative velocity of the droplets decreased, and the inertial force decreased. Under the obstruction of surface tension, the droplets spread slowly and showed a stable trend. On the pear leaves with the inclination angle of 30°, the liquid phase distribution is consistent with that on the pear leaves with the inclination angle of 0° before 0.4 ms. The liquid phase distribution extended from the centre to both sides. After 0.6 ms, the liquid phase distribution tends to shift to the left; however, the droplets eventually spread evenly along both sides. In the first 0.6 ms, the droplets spread evenly along both sides of the pear leaves at inclination angles of 45° and $60^\circ$. After 0.8 ms, the droplets glided in the horizontal direction, and the liquid phase distribution was on the left side. However, the droplets spread on both sides owing to the vertical inertial force.

**Fig. 10:**
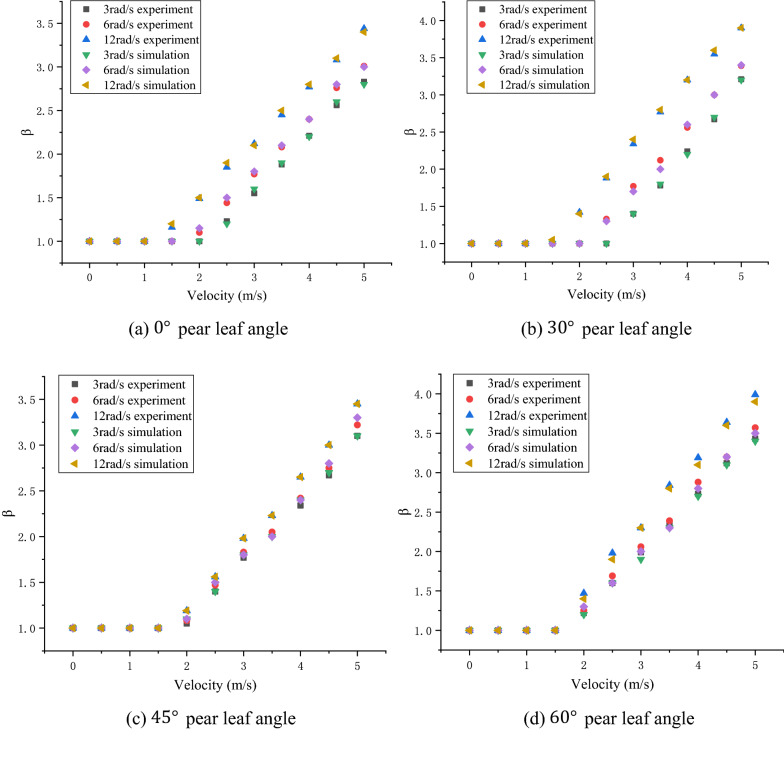
Comparison of experimental and simulation results for 0.1-μL droplets and pear leaves

**Fig. 11:**
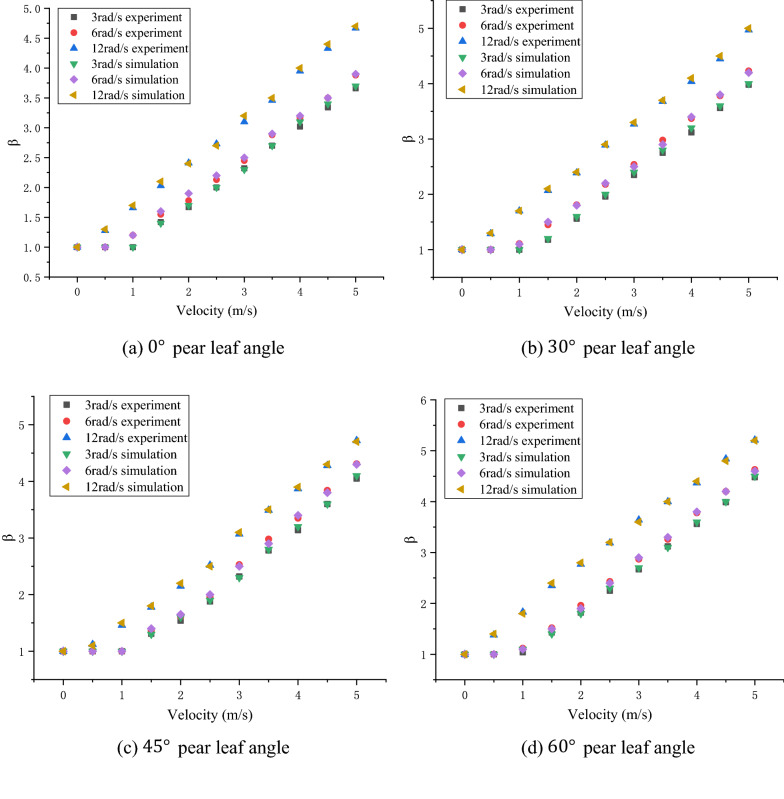
Comparison of experimental and simulation results for 0.5-μL droplets and pear leaves

**Fig. 12:**
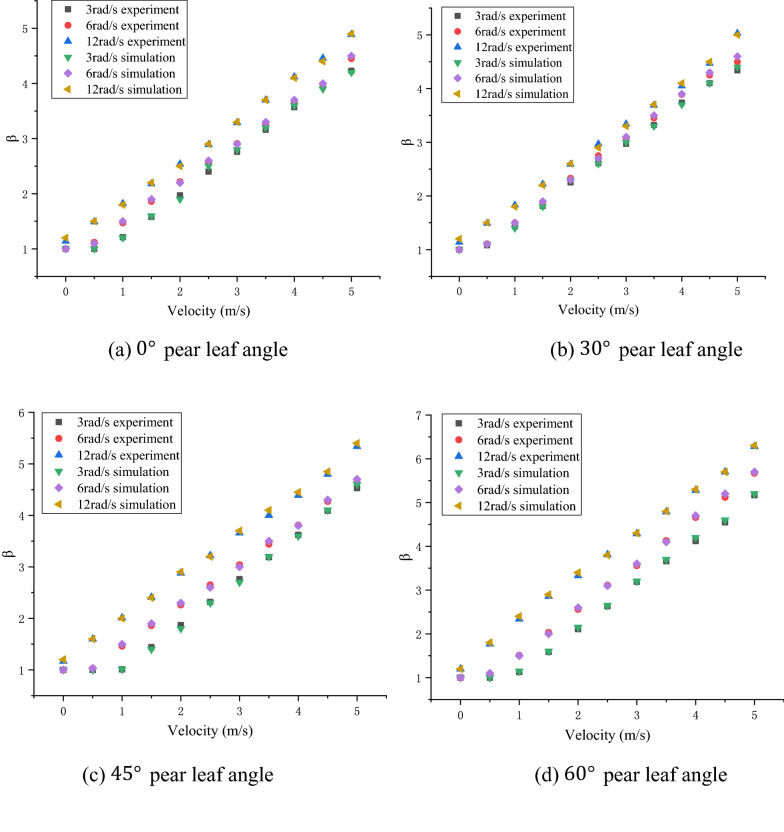
Comparison of experimental and simulation results for 1-μL droplets and pear leaves

In Figs. 10, 11, and 12, the experimental and simulation results of the droplet impact on a pear leaf are compared. The results show that the deviation of the longitudinal spreading factor between the experimental and simulation results was less than 5%. The data reveal that the simulation results are in good agreement with the experimental results.

### Effect of pear leaf vibration on droplet deposition behaviour

Currently, there are few studies on the influence of leaf vibration on droplet deposition behaviour. It is considered that the droplet coverage rate is affected by the aerodynamic response velocity of a leaf under the condition of a given spray liquid and texture of the leaf surface. The coverage rate of the droplets first increased and then decreased with the response velocity of the leaf. Based on the droplet deposition analysis, the uniformity of the quantity distribution and droplet size on the surface of the leaf is related to the aerodynamic response velocity of the leaf. Studies have also been conducted on the classification of leaf vibration, and the deposition behaviour of droplets was analysed assuming a definite leaf vibration type [35]. In this study, the effects of the velocity and angle parameters on the droplet deposition behaviour were analysed.

**Fig. 13:**
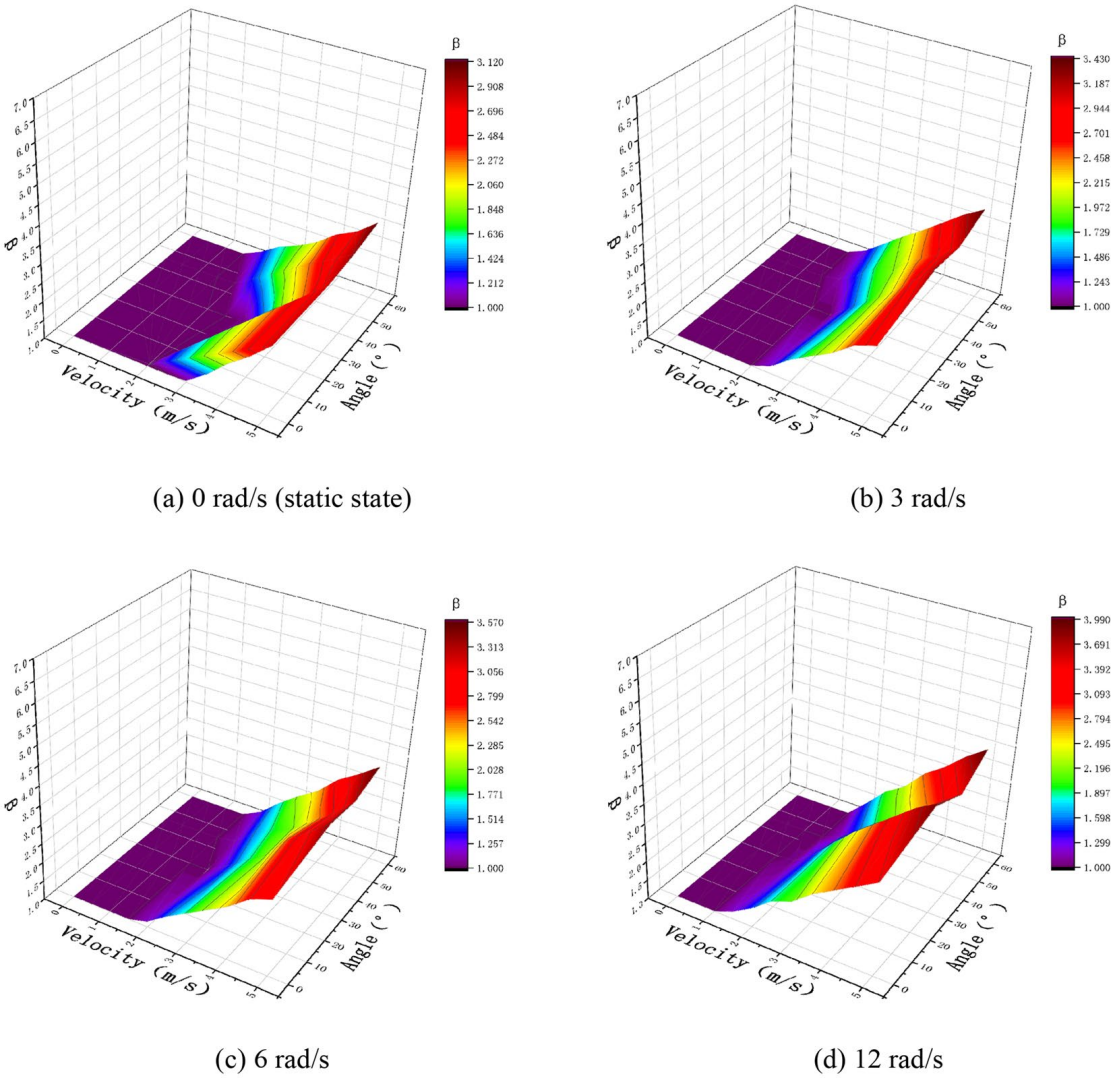
Comparison of the deposition effect of 0.1-μL droplet under static and dynamic conditions

**Fig. 14:**
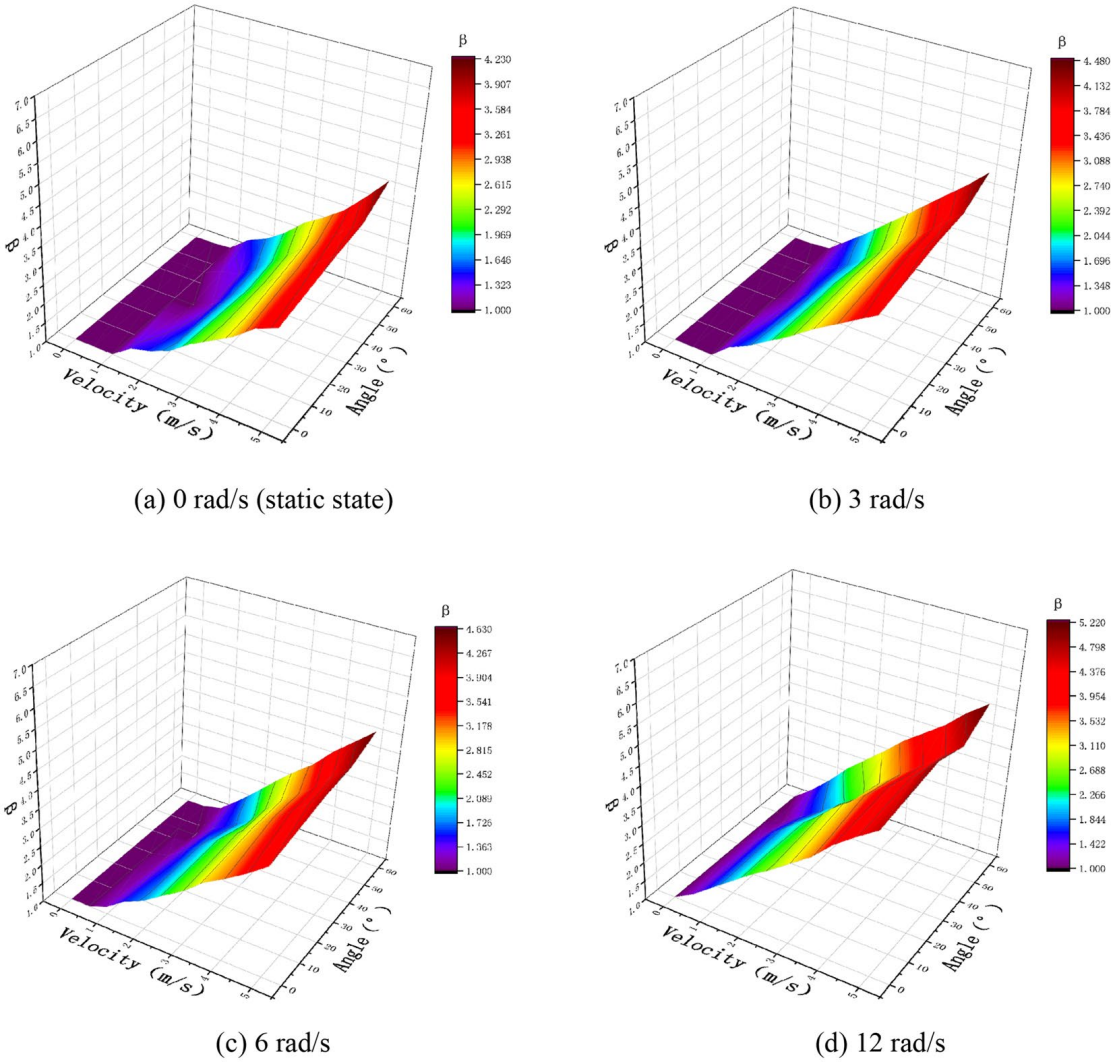
Comparison of the deposition effect of 0.5-μL droplet under static and dynamic conditions

**Fig. 15:**
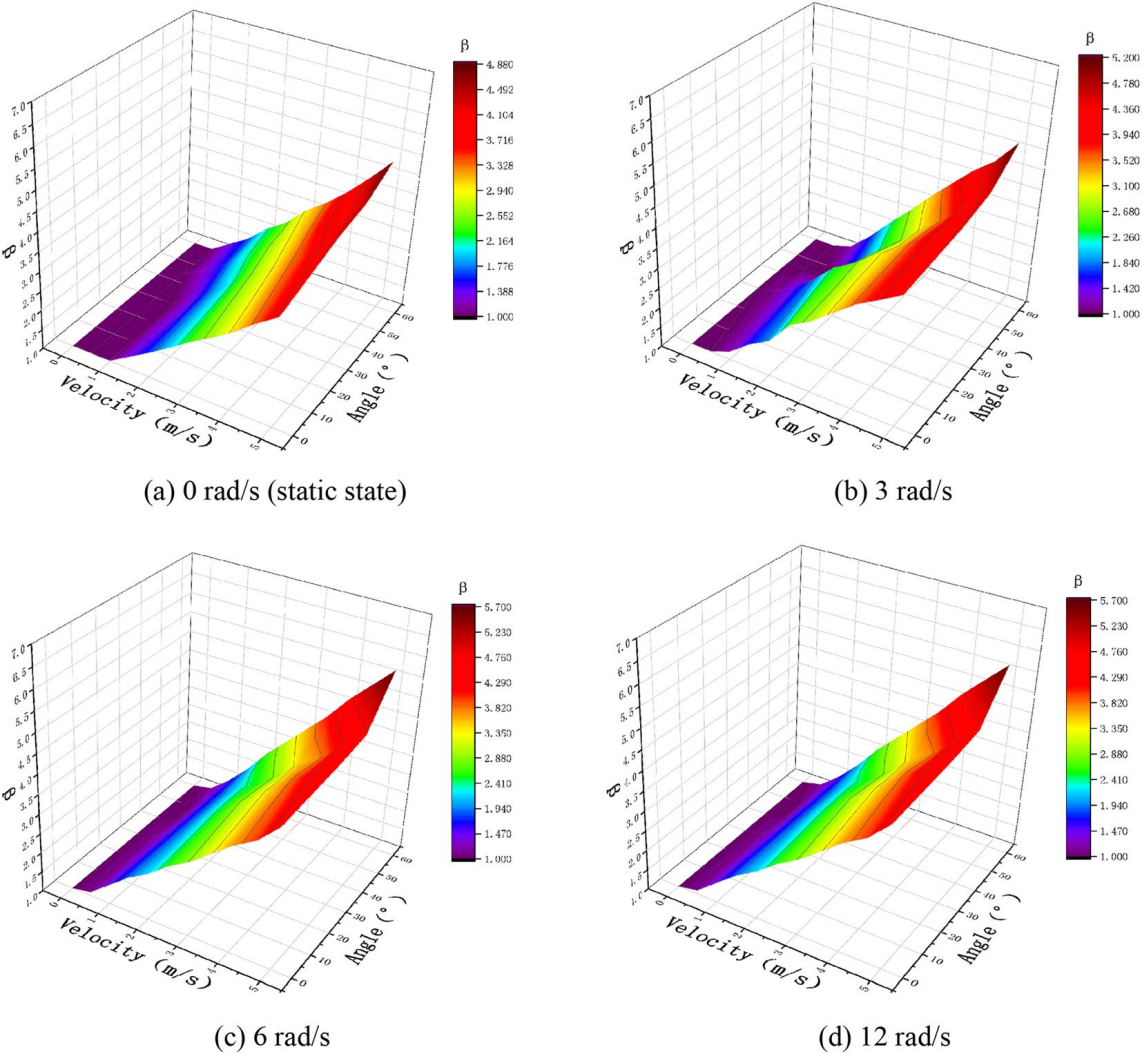
Comparison of deposition effect of 1-μL droplet under static and dynamic conditions

As shown in Figs. 13a, 14a, and 15a, when the droplet collides with the static pear leaf, it does not spread at a low velocity. With the increase in droplet velocity, the longitudinal spreading factor of the droplet increased approximately linearly. Compared with 0.1-μL droplets, the spreading velocities of the 0.5-μL and 1-μL droplet were smaller, and the spreading factor was larger, indicating that larger droplets have prominent instability. According to the theoretical analysis of 3.1, the angle of the leaf determines the vertical and horizontal velocity components, and further affects the spread and slip of the droplets. The increase in leaf inclination can increase the upper limit of the spreading factor of droplets; however, the spreading effect of the low-velocity droplets was not significant when they collided with $30^\circ$ and $45^\circ$ leaves.

In this study, the main variables of pear leaf vibration included angular velocity and leaf angle during collision. As shown in Figs. 13, 14, and 15, an increase in droplet velocity and angular velocity significantly improves the longitudinal spreading of droplets with the same volume. During a collision, an increase in the angular velocity causes the relative velocity to increase. Under the same conditions, the greater the relative velocity, the greater the inertial force. Due to the increase in the inertial force, droplets are unable to maintain their original state; hence, the spreading factor of droplets can be improved in general, thus enhancing the deposition condition of small droplets; however, it also intensifies the instability of large droplets.

### Critical spreading velocities of three droplet types

The vertical (horizontal) critical velocity of a droplet is the minimum velocity of the droplet spreading in the vertical (horizontal) direction when it collides with a vibrating pear leaf. The direction of the velocity of the vibrating pear leaves is consistent with that perpendicular to the pear leaves; thus, the angular velocity is related to the vertical critical velocity of the droplets. Nevertheless, it is independent of the horizontal critical velocity of the droplets.

**Fig. 16:**
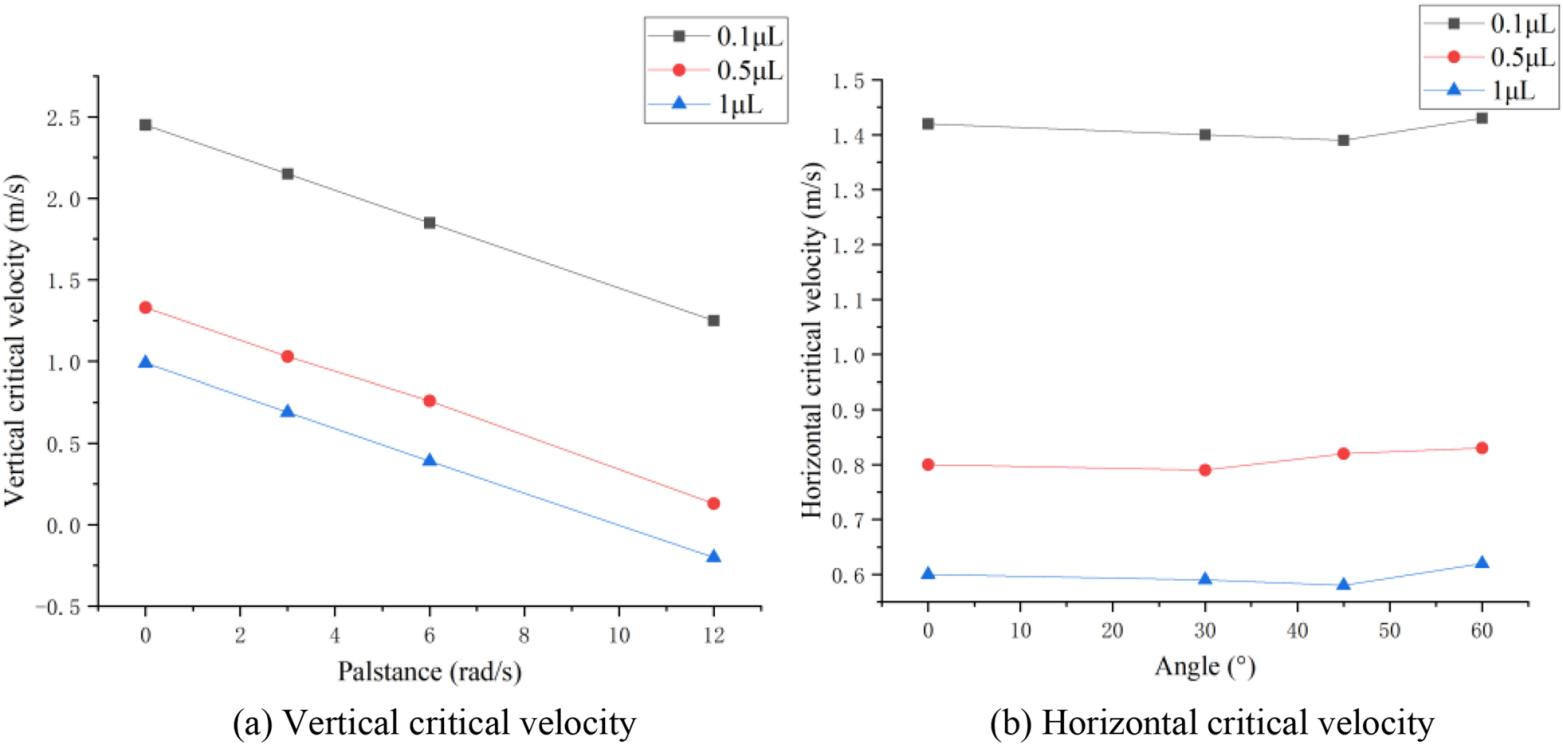
Critical droplet velocity

As shown in Fig. 16a and b, with an increase in the angular velocity of the vibrating leaf, the critical velocity of droplets of different sizes in the vertical direction decreases linearly; however, with the change in the leaf inclination, the critical velocity of the droplets in the horizontal direction of the leaf remains unchanged. However, the critical velocities of 0.5-μL and 0.1-μL droplets in both the vertical and horizontal directions are lower than those of 1-μL droplet, so large droplets spread easier in low-velocity collisions. For the vertical critical velocity of droplets, the increase in the vibration angular velocity of pear leaves significantly reduces the velocity needed for droplet spreading, which indicates that the opposite collision between pear leaves and droplets is beneficial to droplet spreading. The leaf angle during collision has no significant effect on the critical horizontal velocity of the droplets; however, it changes the velocity component of the droplets in the horizontal direction, which can also promote the horizontal spreading of droplets to a certain extent.

In the actual application process, the particle size of mechanically atomised droplets is usually between 50 and 600 μm. The 0.1-μL droplet is quite close to the actual condition of the spray. According to Fig. 13, the absolute velocity of droplet spreading is 2.84 m/s under non-vibration conditions. As shown in Table 1, when the incoming velocities are 5, 6–7, and 9–10 m/s, the corresponding rotation angular velocities are 3, 6, and 12 rad/s, respectively, and the absolute velocities corresponding to the droplet are 2.57, 2.31, and 1.90 m/s respectively. Therefore, the minimum droplet velocity in the actual spray process should not be less than 1.9 m/s. Meanwhile, compared with the critical velocity of a 0.1-μL droplet in the vertical and horizontal directions, the droplet spread more easily in the horizontal direction. Maintaining a certain collision angle will help the spread of droplets; however, an excessive collision angle will reduce the number of droplets colliding with the leaves. The vertical collisions between droplets and plant leaves should be reduced.

In air-assisted spraying machinery operation, the velocity of droplets reaching the canopy should not be less than 2 m/s. When the droplet velocity is too low to interact with the leaf, the situation is similar to that under static adhesion. The droplet is spherical and unstable, which is often not conducive to the deposition and spreading of droplets. In this study, it was easy to observe that only a small spread occurs after droplets break through the critical velocity at the moment of impact. To obtain a larger spread range, a larger relative velocity between the droplets and the leaf, and a reasonable collision angle between the leaf and the droplets are required.

The interaction canopy against droplets is an essential aspect of the spraying process. The collision between droplets and plant leaves is related to the properties of the droplets, characteristics of plant leaves, and relative motion between them [36–38]. At present, most studies only focus on the deposition behaviour of stationary target leaf surface [39–42]. Similar to previous work, velocity, contact angle and the initial inclination angle are taken as the factors affecting the deposition effect. Previous studies have shown the deposition condition of droplets impacting the leaves, which have a certain meaning for the guidance of pesticide application. It is based on the deposition characteristics of droplet collisions with leaf targets to evaluate the deposition effect of droplets. Although previous studies on static leaves can reflect the deposition and spread of droplets on leaves, in the actual air-assisted application process, the air-mist flow would inevitably disturb the leaves before reaching them [43]. Thus, this disturbance of air-mist flow onto leaves will affect the final deposition state of droplets on leaves. Therefore, the theoretical model of the droplet deposition behaviour of static leaves is not suitable for dynamic leaves. These findings and conclusions provided good ideas and methods for our research. This paper studied the deposition characteristics of droplets under vibrating pear leaves by establishing the collision mechanical model and slip mechanical model between droplets and vibrating pear leaves. The collision process between droplets and dynamic pear leaves was simulated in CFD, and the interaction platform between droplets and dynamic pear leaves was built for experimental verification. The experimental results are in good agreement with the simulation results, but there are still areas that need to be improved. The team should also focus on the following issues in the future:

1. The established CFD model only considers the possible results after colliding with a single droplet with pear leaves of different sizes. In the actual air-assisted application process, droplets always act on the surface of pear leaves in the form of droplet groups, so it is necessary to explore the CFD model applicable to droplet groups.
2. For the established leaf vibration model, this study only considered the vibration of the petiole and did not consider rotation of the leaf. Therefore, it is necessary to establish a mechanical model that combines the vibration and rotation of a leaf to study the deposition behaviour of the leaf under wind-induced vibration.

In summary, the CFD model established in this study can reflect the deposition behaviour of droplets on pear leaves under wind-induced vibration to a certain extent, as well as clarify the influence of droplet velocity, leaf inclination angle, and leaf vibration angular velocity on the droplet deposition behaviour within a certain range. However, the model established under ideal conditions and the adaptability of the model need to be further explored. Based on this study, we can further explore the velocity conditions of droplet sliding and splashing, the CFD model suitable for multiple droplets, and the leaf vibration model combining petiole vibration and leaf rotation to enrich the atomisation mechanism and basic application theory of pesticide application technology in orchards, as well as provide references for the study of droplet deposition characteristics under the vibration of fruit trees.

## Conclusions

In this study, a mechanical model of the droplet impact on vibrating horizontal leaves and a sliding model of droplet impact on vibrating inclined leaves were established. The relationship between the change in droplet deposition behaviour and inertial force at the time of droplet impact was discussed. The inertial force generated by the relative velocity was the main reason for the droplet spreading and sliding.

The increase in droplet velocity and angular velocity significantly improved the longitudinal spreading of the droplets. During a collision, an increase in the angular velocity causes the relative velocity to increase. Under the same conditions, the greater the relative velocity, the greater the inertial force. Due to an increase in the inertial force, the droplets are unable to maintain their original state. Thus, the spreading factor of droplets can be improved as a whole, which improves the deposition conditions of small droplets. However, it also aggravates the instability of large droplets, making them prone to coalescence and rolling off. At the same angle, the longitudinal spread range of droplets on the dynamic pear leaves was larger than that under the static condition, and more droplets could spread on the plant leaves. The angle of the leaf determines the vertical and horizontal velocity components, and further affects the spread and slip of the droplets. Maintaining a certain collision angle will facilitate the spread of the droplets. At a minimum, the vertical collisions between the droplets and plant leaves should be reduced. The critical velocities of 0.5-μL and 0.1-μL droplets in both the vertical and horizontal directions are lower than those of 1-μL droplet, so it is easier for large droplets to spread in low-velocity collisions and shows that the larger droplet has prominent instability. Pear leaf vibration can improve the deposition of low-velocity droplets and small droplets, but there is a risk of large droplet loss.

This study provides a simulation method for collisions between a vibrating leaf and moving droplets. The results show that the absolute velocity of droplet spreading is 2.84 m/s under non-vibration conditions. When the incoming velocities are 5, 6–7, and 9–10 m/s, the corresponding rotation angular velocities are 3, 6, and 12 rad/s, and the absolute velocities corresponding to the droplet are 2.57, 2.31, and 1.90 m/s. Therefore, the minimum droplet velocity in the actual spray process should not be less than 1.9 m/s.

## Data Availability

The datasets used and/or analyzed during the current study are available from the corresponding authors upon reasonable request.
